# Retroperitoneoscopic nephrectomy for crossed-fused ectopic kidney

**DOI:** 10.4103/0970-1591.56182

**Published:** 2009

**Authors:** Pranjal Modi, S. J. Rizvi, Rahul Gupta, Suhag Patel

**Affiliations:** Department of Urology and Transplantation Surgery, Institute of Kidney Diseases and Research Centre, Institute of Transplantation Sciences, Civil Hospital Campus, Asarwa, Ahmedabad, Gujarat, India

**Keywords:** Anomaly, kidney, laparoscopy, nephrectomy, retroperitoneoscopy

## Abstract

A 25-year-old female presented with a history of recurrent urinary tract infection and end stage renal failure. Voiding cystourethrography revealed bilateral Grade IV vesicoureteral reflux with left to right crossed ectopia. A computed tomography scan showed fusion of both kidneys with the left kidney situated at the lower and anterior part of the right orthotopic moiety. A retroperitoneoscopic nephrectomy with a right side ureterectomy was carried out.

## INTRODUCTION

Crossed fused ectopia of the kidney is a rare malformation occurring in 0.05% to 0.1% of the population.[1] Approximately 90% of the time, the crossed kidney is fused with the kidney in an orthotopic position. The ureter of the orthotopic kidney usually enters the bladder on the ipsilateral side and the ureter of the ectopic kidney crosses the midline at the pelvic brim and enters the bladder on the contralateral side. Though native renal units are normal in most cases, associated abnormalities like cystic dysplasia, obstruction, vesicoureteral reflux, infection, urolithiasis, twisting, or volvulus may present. We report a case of retroperitoneoscoopic nephrectomy for reflux nephropathy in crossed fused ectopia with recurrent urinary tract infection requiring a nephrectomy prior to transplantation.

## CASE REPORT

**Figure 1:**
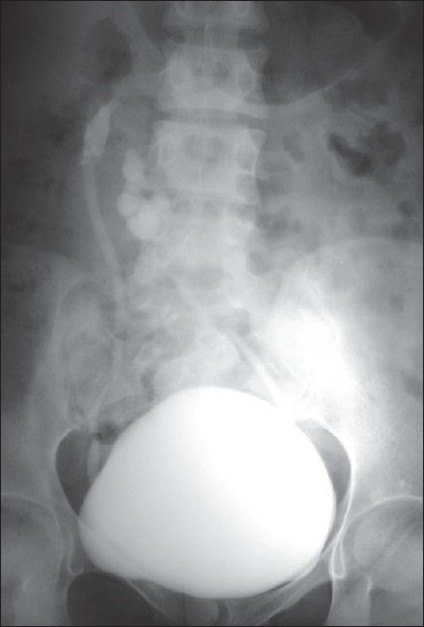
A cystogram showing bilateral Grade IV vesicoureteral reflux and left ureter crossing to the right side at the level of the pelvic brim

**Figure 2:**
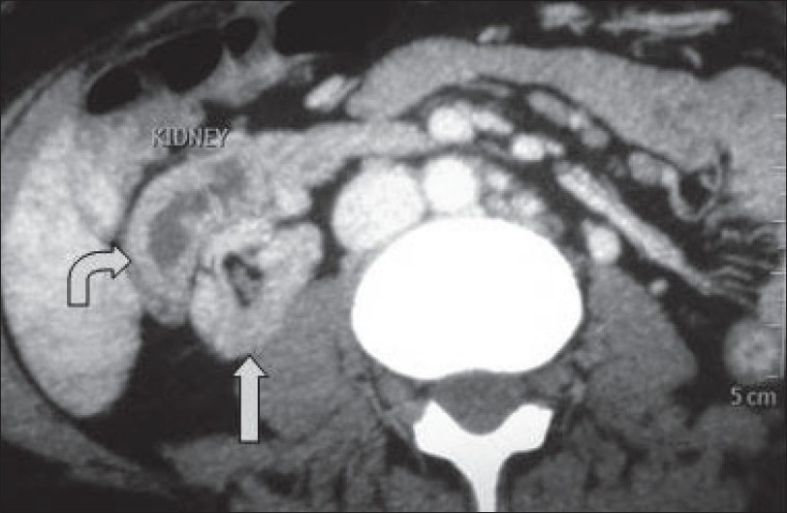
A computed tomography showing crossed fused ectopia. The ectopic kidney is situated anterolateral to the orthotopic kidney

A 25-year-old female with end-stage kidney disease was referred by a nephrologist for the purpose of transplantation. She had a history of recurrent urinary tract infection. A urine analysis was showed microscopic pyuria and a urine culture showed traces of the *E. coli* organism. After treatment with antibiotics, a voiding cystourethrography was performed showing bilateral Grade IV vesicoureteral reflux [Figure 1]. A ultrasonography had detected an empty left renal fossa; an echogenic irregular kidney with an abnormal shape was present in the right lumbar region. A three-dimensional computed tomography (CT) scan with contrast showed a left to right crossed fused renal ectopia with right kidney malrotation. The ectopic fused kidney was situated anterior and lateral to the orthotopic kidney [Figure 2].

After discussing the risks and benefits of open and laparoscopic surgery with the patient, and with consent for open conversion if required, the patient was subjected for retroperitoneoscopic nephrectomy for fused kidneys.

An endotracheal intubation was performed after general anesthesia. The patient was positioned in a flank position and the retroperitoneum was accessed by open technique. The retroperitoneum was accessed by a 1.5 cm long incision at the tip of the 12^th^ rib. Balloon dilation of retroperitoneum was created and a 10 mm port was placed for laparoscope. 10 mm working ports were placed at the angle between the erector spina muscle and the lower border of the 12^th^ rib and a 5 mm port was placed at the anterior axillary line under the guide of a 30° laparoscope. Gerota's fascia was opened by sharp dissection and a dissection on the posterolateral aspect of the inferior vena cava was carried out to find renal vessels. A total of 6 renal arteries and 4 renal veins were identified. The arteries were clipped separately by hem-o-lok clips and divided. The fused kidneys were mobilized completely and both ureters were identified. After mobilization of the fused kidneys and confirming that no additional blood supply was present, the renal veins were clipped and divided. The ureter going to the left side was divided after clipping it with hem-o-lok clips. The specimen was delivered intact by making a small incision (5 cm) in the inguinal region and a complete ureterectomy was carried out on the right side. The wound and port sides were closed.

The operative time was 148 minutes and blood loss was 35 ml. The patient resumed liquids orally 8 hours after surgery and remained in the hospital for 4 days. She is now on maintenance hemodialysis.

## DISCUSSION

Crossed renal ectopia is a rare congenital malformation and is the second most common fusion anomaly after horseshoe kidney. This anomaly occurs more often in men (3:2) and is usually left to right ectopia.

Multiple configurations have been described, including inferior and superior displacement, L-shaped kidney, lump kidney, and sigmoid kidney. In most cases, the kidney fails to ascend and rotate completely. Vesicoureteral reflux is associated with 20% of the cases of crossed renal ectopia and is the most common anomaly encountered.[2] Patients may have recurrent urinary tract infections that present early in childhood. Our patient had developed end-stage renal disease due to reflux nephropathy.

Regardless of the type of fusion encountered, the vascular supply to each kidney is variable and unpredictable. The crossed ectopic kidney is supplied by one or more branches from the aorta or common iliac artery. Therefore, preoperative renal angiography, CT angiography, or magnetic resonance imaging angiography is essential for planning surgical dissection.

Laparoscopic upper moiety and lower moiety for hydronephrotic crossed fused renal ectopia has been described previously.[3,4] Both cases were carried out transperitoneally. A retroperitoneoscopic approach for nephrectomy for crossed fused ectopia has not been previously described. The advantage of retroperitoneoscopic surgery is early vascular access without mobilizing the kidney. Further, in the set up of end-stage renal disease, both fused kidneys are small in size and hence working space in the retroperitoneum is not a problem. The location of the fused kidneys is above the pelvic brim and port placement sites are as for the standard retroperitoneoscopic nephrectomy for an orthotopic kidney. The basic but critical point is identifying and securing all the renal arteries before controlling renal veins to avoid congestion of the kidney and bleeding. In our case, four vessels were arising from the right side common iliac artery and two from the aorta. Unfortunately, in the set up of small size of both the kidneys and end-stage kidney disease, we could not obtain good vascular anatomy in the CT angiogram. In such situations, identification of the vessel during surgery is challenging and careful dissection all around the kidney during its mobilization should be carried out to avoid inadvertent injury to the vessels leading to hemorrhage.

Our case requires nephrectomy for preparation for kidney transplantation. We have performed complete ureterectomy on the right side and removed the specimen from 5 cm long modified Gibson's incision. On left side, the stump of the ureter was planned to remove at the time of left iliac fossa transplantation.

In conclusion, a retroperitoneoscopic nephrectomy for crossed fused ectopia in a patient with end-stage renal disease is feasible and safe. To our knowledge, this is the first case report of a nephrectomy for crossed fused ectopic kidney performed retroperitoneoscopically.
